# Overexpression and biological function of PRDX6 in human cervical cancer

**DOI:** 10.7150/jca.39892

**Published:** 2020-02-10

**Authors:** Xiaoli Hu, Ermei lu, Chunyu Pan, Yichi Xu, Xueqiong Zhu

**Affiliations:** Department of Obstetrics and Gynecology, the Second Affiliated Hospital of Wenzhou Medical University, Wenzhou 325027, China.

**Keywords:** PRDX6, cervical cancer, proliferation, migration, invasion, apoptosis

## Abstract

**Background**: Our previous study demonstrated that the peroxiredoxin 6 (PRDX6) protein was downregulated in squamous cervical cancer samples after neoadjuvant chemotherapy compared with the expression level before chemotherapy. However, the effect of PRDX6 on the biological behavior of cervical cancer is still uncertain. Thus, the purpose of this study was to explore the functional impacts of PRDX6 gene on the biological behavior of cervical squamous cancer cells.

**Methods**: An immunofluorescence assay was applied to evaluate the expression difference of PRDX6 between cervical cancer tissue and normal cervical tissue samples. A lentivirus was used to upregulate and downregulate PRDX6 expression in SiHa cells. Furthermore, the role of PRDX6 on cell proliferation, apoptosis, migration and invasion was evaluated. Additionally, the effect of PRDX6 on the progression of the cervical cancer was investigated via a xenograft model in BALB/c nude mice that either overexpressed or underexpressed PRDX6.

**Results**: The expression of PRDX6 was generally increased in cervical cancer tissues. Furthermore, the overexpression of PRDX6 stimulated the proliferation, migration and invasion of cervical squamous cancer cells, and suppressed cell apoptosis. The opposite results were also obtained after successful knockdown of PRDX6. In addition, the overexpression of PRDX6 significantly promoted the growth of cervical carcinoma* in vivo*.

**Conclusions**: PRDX6 promoted the proliferation, migration and invasion, and inhibited apoptosis in cervical cancer cells, indicating that PRDX6 is an important promoter of cervical cancer tumorigenicity.

## Introduction

Cervical cancer remains one of the most frequently diagnosed malignancies and is still the second leading cause of cancer deaths among females worldwide 1, 2. Despite the rapid development of preventions and cures for cervical cancer, the overall prognosis for patients with advanced or recurrent cervical cancer is still discouraging 3. Therefore, many researchers are engaged in identifying additional molecular regulators, which would likely contribute to improving the prognosis of cervical cancer.

A number of reactive oxygen species (ROS), including hydrogen peroxide (H_2_O_2_), hydroxyl radical (^.^OH), superoxide radical (^.^O^2^-) and oxygen (O^2^), are found to be the byproducts of normal metabolism 4. Recent studies suggest that ROS play an important role in signal transduction messengers, which are linked to cellular transformation, inflammation, tumor survival and so on 5. There are excessive accumulation of ROS in cancer cells compared with normal cells, resulting in the molecular, biochemical and physiological changes necessary for cancer initiation and progression as well as resistance to radiotherapy and chemotherapy 6. In cells, superabundant ROS are suppressed by various antioxidant systems, including PRDX/TRX and GPX/GSH 7.

Peroxiredoxins (PRDXs), a universal superfamily of antioxidant enzymes, are thought to catalyze the reduction of all kinds of cellular peroxides to protect cells against oxidative damage 8. In addition to their role in oxidant detoxification, PRDXs also play a significant role in cell proliferation, differentiation, apoptosis, and redox-mediated signal transduction. There are six members of the PRDX family in mammals, PRDX1-6, which are divided into two classes: 2-Cys PRDXs and 1-Cys PRDXs 9. Among them, only PRDX6 has a single conserved Cys residue leading to a different catalytic cycle, and it utilizes glutathione rather than thioredoxin as the electron donor 10. PRDX6 is highly expressed in a variety of cancer cells, including lung, ovarian, tongue, and breast cancer 11-14. PRDX6 has been recently proven to be a tumor promoter in a variety of cancers. In these carcinomas, overexpression of PRDX6 stimulates cancer cell proliferation and the aggressive phonotype 15, 16. Additionally, Yun et al. found that PRDX6 contributed to the lung tumor growth by increasing the activities of glutathione peroxidase and iPLA2 17. However, there was little information about the effect of PRDX6 in the human cervical cancer. In our previous study, we found that the expression level of PRDX6 was significantly downregulated in cervical cancer tissues after neoadjuvant chemotherapy through proteomic technology, indicating that it may play an important role in chemotherapy of cervical cancer patients 18. However, the role of PRDX6 in cervical cancer progression and metastasis has not yet been reported.

Thus, the purpose of this study was to explore the functional impacts of the PRDX6 gene on the biological behavior of cervical squamous cancer cells. In the present study, we regulated the expression level of PRDX6 in SiHa cells to evaluate the proliferation, apoptosis, migration and invasion capacities of cervical cancer cells. Moreover, we investigated whether PRDX6 would affect the growth of tumors through *in vivo* experiments.

## Methods and Materials

### Patients and tissues collection

Samples of 20 pathological tissues with cervical cancer and 10 tissues from normal cervixes were collected in the Second Affiliated Hospital of Wenzhou Medical University, Wenzhou, China. This study was approved by the Board and Ethical Committee of Wenzhou Medical University. Written informed consent was acquired from all patients who participated in this study in accordance with the Declaration of Helsinki.

### Immunohistochemical staining

All tumor tissues were fixed in formaldehyde and paraffin-embedded for further analysis. Immunohistochemical (IHC) was performed using the streptavidin-biotin complex (SABC) method as described previously 19. The following antibodies were used in the IHC assay: rabbit polyclonal antibody to PRDX6 (1:1000, ab59543, Abcam) and mouse monoclonal antibody to PCNA (1:500, ab29, Abcam). IHC stained pictures of the slides were captured on a light microscope (Olympus, Tokyo, Japan). The results of staining were evaluated separately by two researchers. The staining intensity was graded as follows: 0, negative staining; 1, weak staining; 2, intermediate staining; and 3, strong staining. The proportion of stained positive cells was graded according to the following criteria: 0 (<5%), 1 (>5%-25%), 2 (26-50%), 3 (51-75%), and 4 (76-100%). The staining index was assessed as the percentage of positively stained tumor cells × staining intensity. In addition, a positive control and negative control were used to ensure the validity of the staining. The selected antibodies, including PRDX6 or PCNA, were replaced by PBS in the negative control group, and a tissue type known to express the protein of interest was used as the positive control.

### Cell lines and cell culture

The human cervical cancer cell lines, including SiHa, HeLa, Caski, MS751 and C33A, were purchased from American Type Culture Collection (ATCC, Manassas, VA, USA). SiHa cells were cultured in Dulbecco's modified Eagle's medium (DMEM) (Invitrogen, NY, USA) supplemented with 10% fetal bovine serum (FBS), 100 U/mL penicillin, and 100 μg/mL streptomycin at 37℃ in a humidified 5% CO_2_ incubator.

### Construction of PRDX6 Lentiviruses

Lentivirus-based particle expressing GFP was produced by transient cotransfection of HEK293 cells with plasmid recombination. First, GFP-PRDX6 cDNA and non-silenced short hairpin RNA (shRNA) were generated *in vitro* by an outside company (Synbio Technologies, Shanghai, China). After identification by gene sequencing, recombinant plasmids including the overexpression vector pLVX-IRES-ZsGreen 1 (donated by the School of Medical Lab Science, Wenzhou Medical University) and knockdown vector PLKO.1 were transfected into human embryonic kidney HEK293 cells. Finally, the lentivirus was obtained by packaging plasmids psPAX2 and the G protein of the vesicular stomatitis virus (VSV-G) envelope plasmid pMD2.G (donated by Dr Luzhe Sun, The University of Texas Health Science Center, San Antonio, USA).

### Overexpression and knockdown of PRDX6

Lentiviruses were harvested and filtered through a 0.45 μm filter to transfect the cervical cancer cells (SiHa). To produce stable high expression cell lines, SiHa cells were infected with lentivirus containing pLVX-PRDX1-IRES-ZsGreen 1. In the low expression group, SiHa cells were transfected with lentivirus containing PRDX6 shRNA to induce sequence-specific silencing of PRDX6. The corresponding empty pLVX-IRES-ZsGreen 1 vector and PLKO.1 vector were applied as negative controls for the high and low expression groups, respectively. The optimization and verification of the transfection efficiency was carried out by Western blot. And the words as control or WT, vector, PRDX6, Sh-Ctrl and Sh-PRDX6 represent blank control, an empty vector control of PRDX6 overexpression, overexpression of PRDX6, an empty vector control of PRDX6 knockdown and PRDX6 knockdown group, separately.

### Western blot

Western blot was used to detect specific proteins and was performed as previously reported 20. The expression levels of PRDX6 in diverse cervical cancer cells were analyzed by western blot method. First, the proteins were extracted from the cells and quantitated by the bicinchoninic acid (BCA) protein assay kit (Thermo Scientific, IL, USA). After separation by sodium dodecyl sulfate-polyacrylamide gel electrophoresis (SDS/PAGE), transfer to a membrane made of polyvinylidene difluoride (PVDF), and blocking of nonspecific binding by 5% skim milk powder, the membrane was incubated overnight at 4℃ with related antibodies, including PRDX6 (rabbit antibody, 1:1,000, ab59543, Abcam), BAX (rabbit antibody, 1:1,000, ab32503, Abcam), Bcl-2 (rabbit antibody, 1:1,000, ab32124, Abcam), Tubulin (mouse antibody, Boster, 1:2,000, BM145) and β-actin (mouse antibody, Boster, 1:2,000, BM5422). After washing in Tris Buffered Saline with Tween 20 (TBST), the blot was incubated with the corresponding secondary antibodies for 2 h at room temperature. The protein expression levels were evaluated by using an Amersham enhanced chemiluminescence (ECL) Western blotting detection kit (GE Healthcare, NJ, USA). The relative protein expression was normalized to the signal intensity of tubulin or β-actin.

### Cell counting kit-8 (CCK-8) assay

Cell proliferation was evaluated using a Cell Counting Kit-8 assay according to the manufacturer's instructions. SiHa cells (1000 cells/well) with different expression levels of PRDX6 were seeded into 96-well plates and cultured for 1, 2, 3, 4, and 5 days. Then, CCK-8 (10 μL/well) was added. After 3 h, the absorbance value was evaluated at 450 nm by a microplate reader (Bio-Rad, USA). These experiments were repeated three times.

### Colony formation assay

A clonogenic assay was further performed to investigate the function of PRDX6 on cell proliferation. SiHa cells with different PRDX6 expression levels at a density of 2000 cells/mL were seeded into 6-well plates. After 4 weeks, the cells were fixed in 4.0% paraformaldehyde for 20 min and then stained with crystal violet for 30 min. The surviving fractions of the clonogenic cells were calculated to assess the effects of different expression levels of PRDX6 on cell proliferation.

### Flow cytometric analysis

To assess the effect of PRDX6 expression on cervical cancer cell apoptosis, a total of 2 × 10^5^ SiHa cells with different expression levels of PRDX6 were seeded into 6-well plates. After incubation for 48 h, the seeded cells were digested with 0.25% trypsin without ethylenediaminetetraacetic acid (EDTA) and dissociated into single cells. Then, the cells were stained with the Annexin V-phycoerythrin (PE) and 7-amino-actinomycin D (7-AAD) apoptosis detection kit (BD, Franklin Lakes, NJ, USA) for 15 min. The percentage of apoptotic cells was evaluated by flow cytometry (Beckman Coulter Life Sciences, USA). The total procedure was repeated three times.

### Wound healing assay

For the scratch wound assay, the SiHa cells (1 × 10^6^ cells per well) were seeded 6-well plates. After 24 h of transfection, a wound was made in confluent cell monolayers using a sterile 200 μL pipette tip, and the cells were then cultured in fresh medium with 1% FBS. The pictures of cell migration into the wound area and recovery of the monolayer were captured at two different time points (0 h, 24 h) by a phase contrast microscope and digitally photographed (Nikon, Tokyo, Japan). Each experiment was repeated independently in triplicate.

### Cell invasion assays

Cell invasion assays were examined in a 24-well transwell chamber (Corning, NY, USA) coated with Matrigel growth factor. A total of 1 × 10^5^ cervical cancer cells in serum-free DMEM were cultured into the upper part of each transwell chamber, and 600 μL of DMEM with 10% FBS was added to the lower chambers. After 24 h, the invading cells were stained with 2% crystal violet solution for 15 min. Then the cells were photographed and counted from five random fields by a light microscope. All assays were repeated independently three times.

### Tumor xenograft model

The use of laboratory animals was approved by the Animal Ethics Committee of Wenzhou Medical University. A total of twenty female BALB/c nude mice (5 weeks old) were acquired from Charles River Laboratories (Beijing, China). They were randomized into four groups including overexpression of PRDX6, PRDX6 shRNA, and their corresponding controls. After 14 d, mice were injected hypodermically with a suspension of the treated SiHa cells (5×10^6^ cells) on the right flanks. Tumor size was evaluated every three days. Tumor volume was calculated with the following formula: tumor volume = length × width^2^/2. The mice were sacrificed after 5 or 6 weeks, and the tumors were separated and weighed.

### TdT-mediated dUTP Nick-End Labeling (TUNEL) assay

Apoptotic cells were assessed by an In Situ Cell Death Detection Kit (Roche, Indianapolis, IN, USA), according to the manufacturer's instructions. Following traditional dewaxing and hydration, the permeabilization was conducted with 0.1% Triton X-100 for 5 min. After washing with phosphate buffer saline (PBS) 3 times, the paraffin sections were then incubated with 100 μL of TUNEL reaction mix at 37℃ for 1 h. Then, the slides were wash three times with PBS, and further covered with DAPI (4',6-diamidino-2-phenylindole) solution for 15 min. At last, a fluorescence microscope was used to take fluorescent pictures and assess the apoptotic cells. The percentage of TUNEL-positive cells was evaluated by counting all cells from 5 randomly chosen fields (× 400 magnification) per slide.

### Statistical analysis

Data are shown as the means ± standard from at least three independent experiments. One-way ANOVA was applied to evaluate the differences among the multiple groups. Student's t-test was used to perform the statistical comparisons between two groups. If the variances were homogeneous, two groups were compared using the least significance difference (LSD) method. Otherwise, Dunnett's T3 method was included to analyze nonhomogeneous variances between two groups. All statistical tests were two-sided, and *P* < 0.05 (*) and *P* < 0.01 (**) were considered statistically significant. All analyses were performed using SPSS version 18.0.

## Results

### PRDX6 expression is upregulated in human cervical cancer

**Figure 1:**
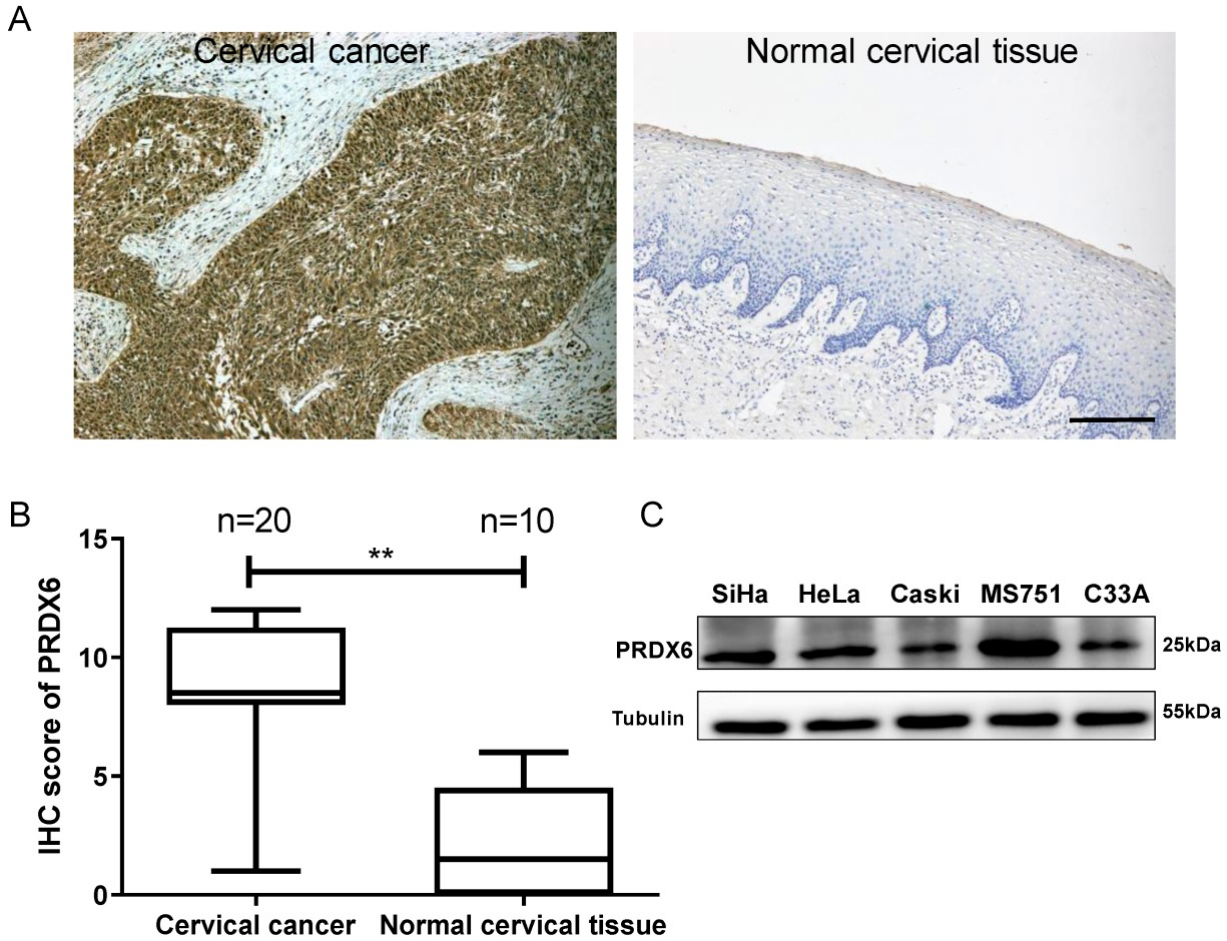
Expression level of PRDX6 in cervical cancer and normal cervical tissue. (A) Immunohistochemical staining of PRDX6 in cervical cancer (T) and normal cervical tissue (N). (B) The IHC scores of PRDX6 expression in normal and cervical cancer tissues. (C) The PRDX6 protein levels in different cervical cancer cell lines, including SiHa, HeLa, Caski, MS751 and C33A. Bar = 200 μm. **P < 0.01.

To explore the role of PRDX6 in human cervical cancer tissues, the expression difference in 20 cervical cancer and 10 normal cervical specimens were compared using the IHC method. As shown in **Figure 1A** and** 1B**, the PRDX6 protein was mainly localized in the cytoplasm of cervical cancer cells and PRDX6 expression was remarkably increased in cancerous tissues compared with normal cervical tissues (7.8 ± 3.4 vs. 2.4 ± 2.4, *P* < 0.01). These results indicated that PRDX6 may play a vital role in the occurrence and development of cervical cancer. The PRDX6 expression level in different cervical cancer cells was analyzed by western blot method. As shown in **Figure 1C**, the highest and lowest expression of PRDX6 were in MS751 and C33A cells, respectively. In addition, PRDX6 protein level in SiHa cell line is moderate among these five cervical cancer cell lines, in which the PRDX6 expression was relatedly easy to be upregulated or downregulated to further explore the biological effect of PRDX6 in human cervical cancer.

### Lentivirus-mediated upregulation and downregulation of PRDX6 in SiHa cells

**Figure 2:**
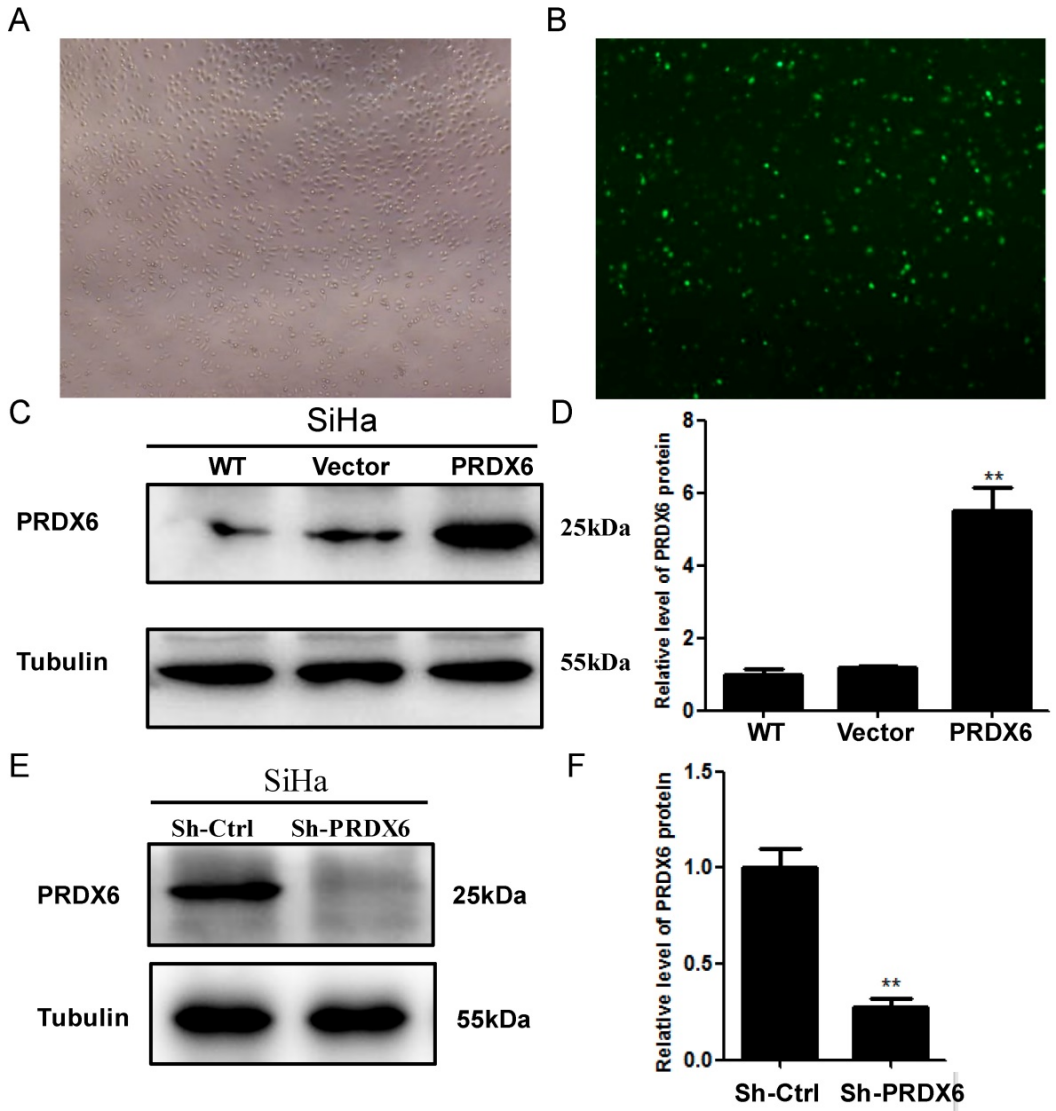
** The transduction efficiency of PRDX6 after transfecting SiHa cells for 48h.** (A) Image of SiHa cells at normal light (× 100). (B) Image of SiHa cells infected with Lentiviruses vector pLVX-IRES-ZsGreen 1 at fluorescent. (C and D) The expression level of PRDX6 protein in SiHa cells transduced with PRDX6 overexpression vector. (E and F) PRDX6 protein expression in SiHa cells transfected with PRDX6 shRNA. Additionally, the names as control or WT, vector, PRDX6, Sh-Ctrl and Sh-PRDX6 represent blank control, an empty vector control of PRDX6 overexpression, overexpression of PRDX6, an empty vector control of PRDX6 knockdown and PRDX6 knockdown group, respectively. Data are expressed as mean ± standard deviation from triplicated experiments. **P < 0.01.

To investigate the biological function of PRDX6 in human cervical cancer cells, SiHa cells were transfected with a lentivirus containing PRDX6 cDNA or PRDX6 shRNA to upregulate or downregulate PRDX6 expression, respectively. The efficiency of the upregulation of PRDX6 through transfection with the lentivirus containing pLVX-PRDX1-IRES-ZsGreen 1 is shown in **Figure 2A** and **2B**. As shown in **Figure 2C** and** 2D**, the expression of PRDX6 in SiHa cells was significantly enhanced through transfection with the lentivirus containing PRDX6 cDNA by approximately 5-fold. PRDX6 expression was effectively suppressed to 25% of the control level by transfection with the PRDX6 shRNA lentivirus (**Figure 2E** and **2F**).

### PRDX6 overexpression promotes cervical cancer cells proliferation

**Figure 3:**
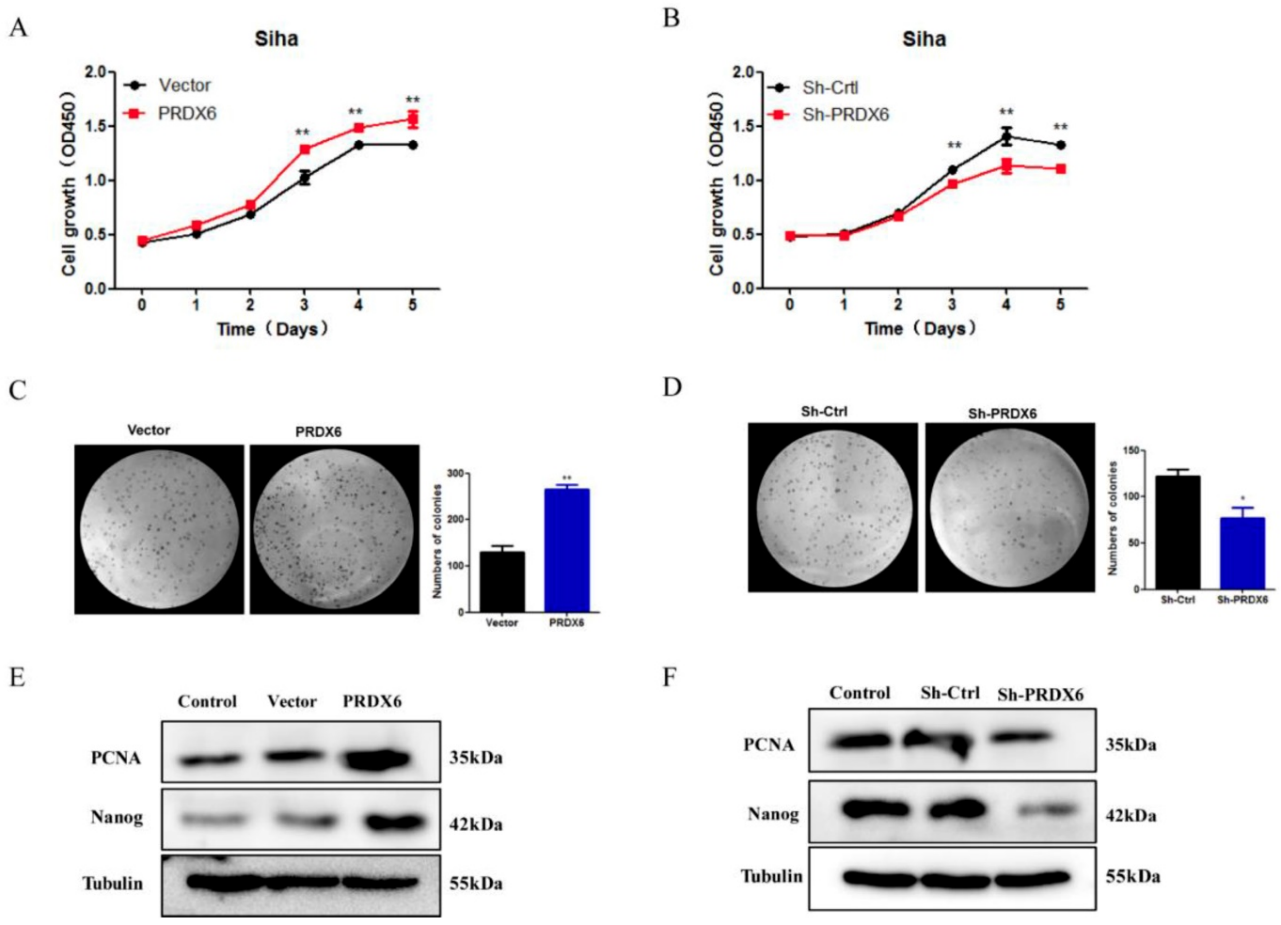
** The influence of PRDX6 on the proliferation of cervical cancer cells.** (A) The effect of PRDX6 overexpression on the proliferation of SiHa cells assessed by CCK-8 assay. (B) The effect of PRDX6 knockdown on the proliferation of SiHa cells. The impacts of PRDX6 overexpression (C) or PRDX6 knockdown (D) on colony formation of SiHa cells. (E and F) The expression of PRDX6, Nanog and PCNA in PRDX6 overexpressed or knockdown SiHa cells were detected by Western blot methods. *P < 0.05, **P < 0.01, data are presented as mean ± standard deviation from triplicate experiments.

To evaluate the effect of PRDX6 on the proliferation and growth of cervical cancer cells, CCK8 assays and colony formation assays were performed to assess the cell viability and growth properties. As shown in **Figure 3A**, the CCK8 assay revealed that the proliferation rate of SiHa cells increased significantly in response to PRDX6 overexpression, especially 3, 4 and 5 d after seeding compared with the empty vector group. The results showed that cell viability was reduced significantly in the PRDX6 shRNA group, indicating that PRDX6 enhances cervical cell proliferation (**Figure 3B**). Additionally, PRDX6 overexpression dramatically stimulated the clone formation of SiHa cells, whereas knockdown of PRDX6 suppressed their ability to form colonies, as indicated by the colony formation assay (**Figure 3C** and **3D**). For instance, there were approximately 360 colonies in the PRDX6 overexpression group when single cancer cells were planted over a 4-week period, which was obviously increasing compared with the control group.

Furthermore, Western blot analysis was used to evaluate whether PRDX6 affects proliferation-related proteins, such as PCNA and Nanog. As shown in **Figure 3E** and **3F**, the expression of PCNA and Nanog protein was remarkably enhanced in the PRDX6 overexpression group. The opposite result was shown in the PRDX6 knockdown group. These results indicated that PRDX6 accelerates the growth of cervical cancer cells probably through upregulation of PCNA and Nanog.

### PRDX6 overexpression suppresses cervical cancer cells apoptosis

**Figure 4:**
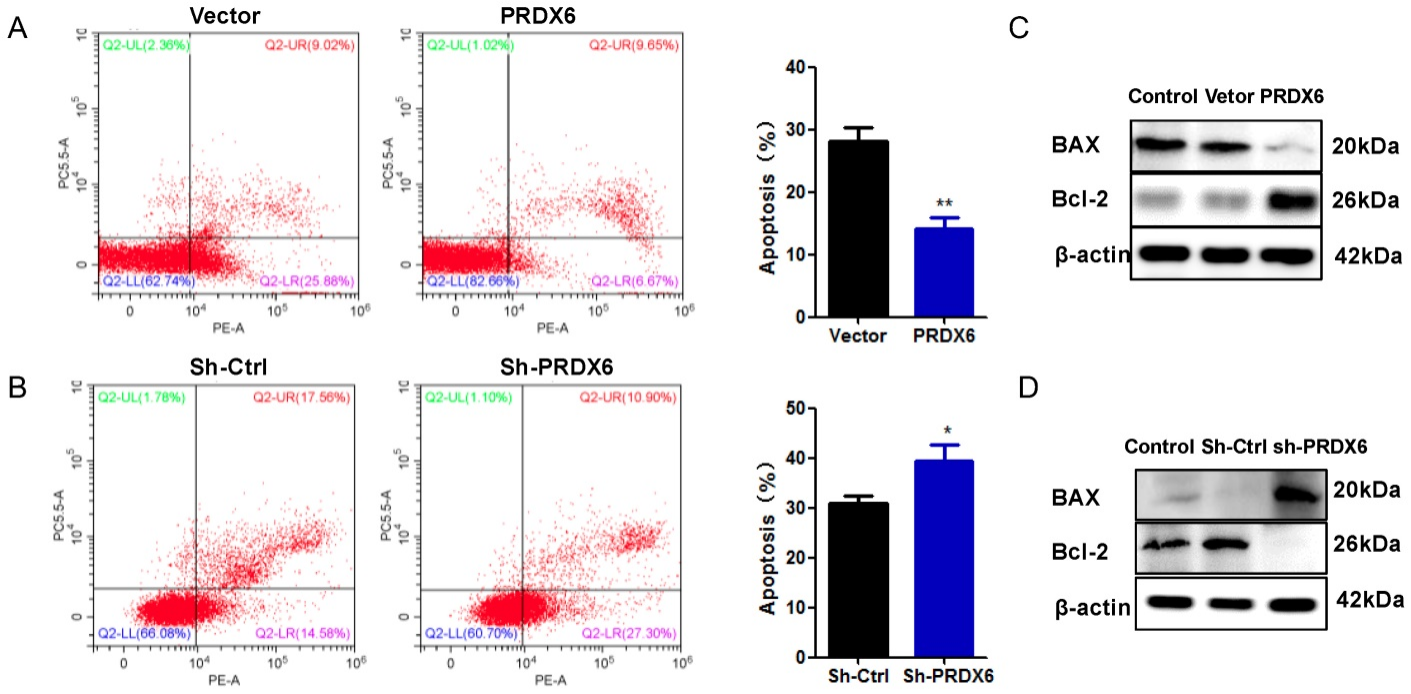
** The effect of PRDX6 on cervical cancer cell apoptosis.** (A) Apoptosis percentage in SiHa cells with PRDX6 overexpression was evaluated by Annexin V-APC/7-AAD staining. Control: cDAN vetor control. (B) Apoptosis rate in PRDX6 knockdown group was analyzed by Annexin V-APC/7-AAD staining. Control: shRNA vetor control. (C and D) The expression levels of BAX and Bcl-2 in SiHa cells with upregulation or downregulation PRDX6 were measured by Western blot method. *P < 0.05, **P < 0.01. Each bar represents mean ± standard deviation from triplicate experiments.

To further explore the influence of PRDX6 on cervical cancer cell survival, the present study measured cell apoptosis using flow cytometry. As presented in** Figure 4A**, the apoptosis rates of SiHa cells overexpressing of PRDX6 were dramatically decreased compared with the empty vector group (*P* < 0.01). As shown in **Figure 4B**, the opposite result was observed in cells with lower expression of PRDX6, in which the average percentage of apoptotic cells was 38.9% in PRDX6 knockdown cells versus 31.2% in vector control cells (*P* < 0.05). The results indicated that PRDX6 silencing resulted in a significant promotion of SiHa cell apoptosis. Then, Western blot analysis was applied to explore the regulatory role of PRDX6 in apoptosis-related protein expression. The results in **Figure 4C** showed that overexpression of PRDX6 significantly decreased the level of BAX and increased Bcl-2 expression, and the ratio of Bcl-2 to BAX was remarkably increased. Conversely, the expression of BAX and Bcl-2 was significantly augmented or inhibited in PRDX6 shRNA group, respectively (**Figure 4D**). Thus, these results demonstrated the anti-apoptotic roles of PRDX6 on cervical cancer cells.

### PRDX6 overexpression promotes the migration and invasion of cervical cancer cells

**Figure 5:**
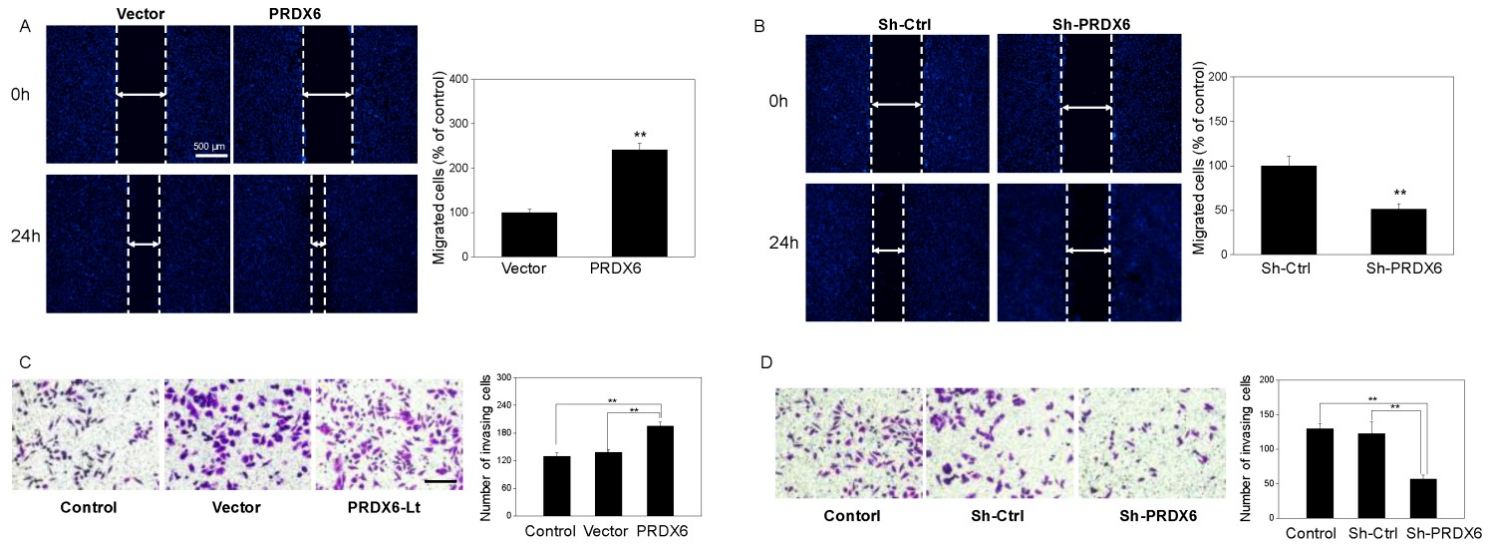
** The influence of PRDX6 on the cell migration and invasion of cervical cancer cells.** (A and B) Cell migration in PRDX6 overexpression or knockdown group was investigated by wound healing assay (×100), separately. (C and D) Cell invasion was assessed by transwell invasion method (× 100) in SiHa cells with upregulation or downregulation of PRDX6 expression, respectively. The control group was the untreated SiHa cells. Data are expressed as mean ± standard deviation from triplicate experiments (**P < 0.01).

To investigate whether PRDX6 upregulation would lead to the migration and invasion of cervical cancer cells, a would healing assay and Matrigel invasion assays were performed. Based on the results of the wound healing assay, SiHa cell migration was remarkably enhanced with PRDX6 upregulation (*P* < 0.01), compared with the blank control group or empty vector group (**Figure 5A**). Conversely, the number of migrated cells per field was significantly decreased in the PRDX6 downregulation group compared to the control or vector groups (*P* < 0.01, **Figure 5B**). Meanwhile, as shown in **Figure 5C**, the number of invading cells significantly increased after PRDX6 overexpression in SiHa cells. In contrary, PRDX6 knockdown obviously reduced the number of invading SiHa cells (**Figure 5D**).

### PRDX6 overexpression promotes tumor growth of cervical cancer cells* in vivo*

**Figure 6:**
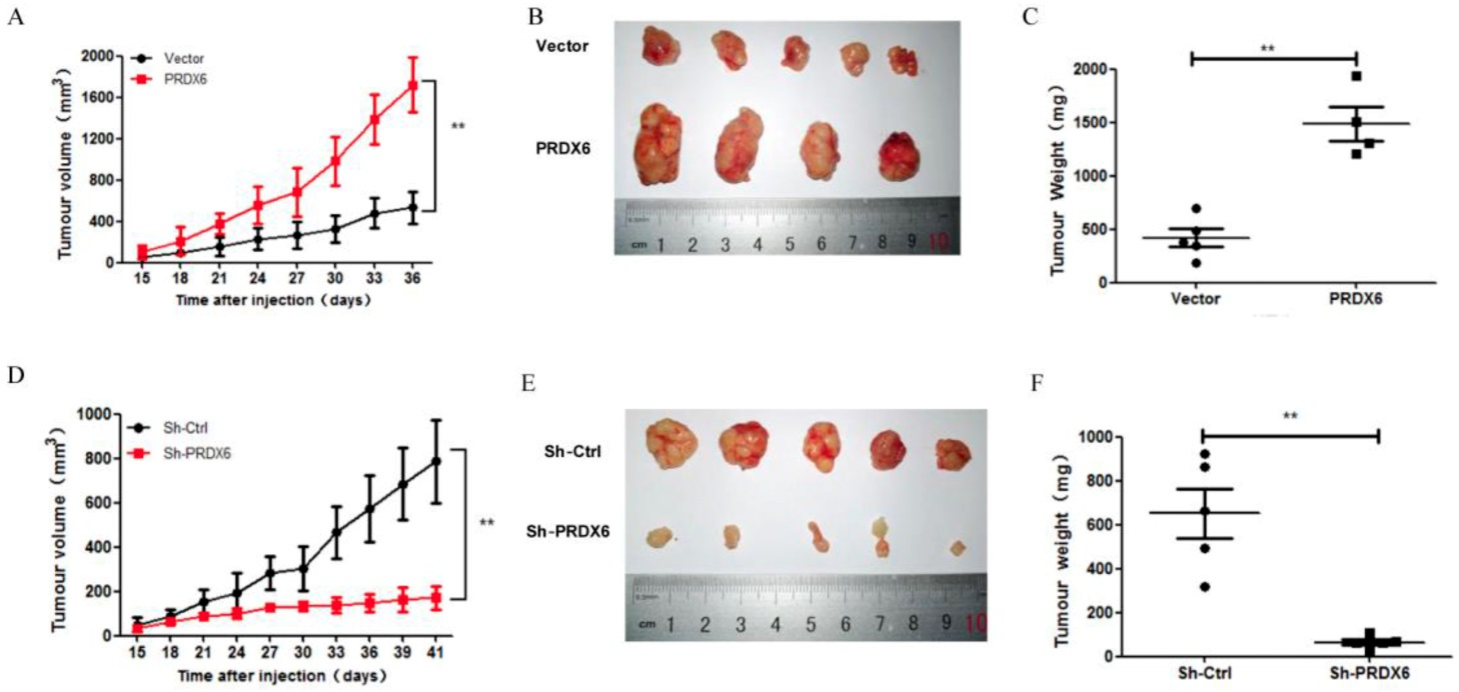
** The effect of PRDX6 on growth of cervical cancer *in vivo*.** (A) Tumor volume of subcutaneous xenograft tumor model developed from SiHa cells with PRDX6 overexpressed. The number of mice in each group is 5, and the length and width of tumor was measured every 3 days. The tumor volumes were presented as mean ± standard deviation. (B) The photographs of tumor from different groups. (C) The tumor weight was evaluated after tumors were harvested. (D, E and F) Tumor volume, cross appearance, and weight in PRDX6 knockdown group *in vivo*, respectively. **P < 0.01.

**Figure 7:**
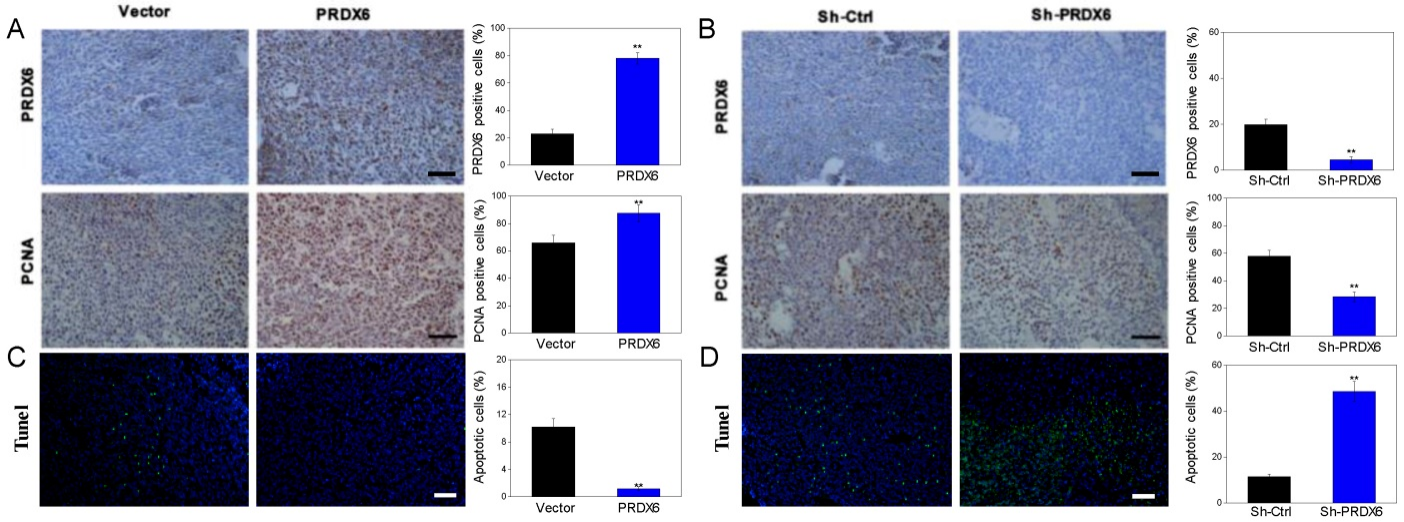
** The expression levels of PRDX6 and PCNA, as well as TUNEL-positive apoptotic cells in xenograft tumor.** (A and B) The PRDX6 and PNCA expression of tumor tissue, which were developed from PRDX6 overexpression or knockdown SiHa cells, were measured by IHC staining, respectively. Bar = 50 μm. (C and D) TUNEL-DAPI co-incubation test of tumor tissue in PRDX6 overexpression or knockdown was used to evaluate the apoptosis cells, respectively. Bar = 50 μm. **P < 0.01.

We further explored whether PRDX6 was involved in the tumorigenesis of cervical cancer *in vivo*, in which a SiHa cell xenograft tumor model was used in the BALB/c nude mice. Consistent with the results* in vitro*, PRDX6 overexpression significantly increased the tumor volume and weight compared to the control (*P* < 0.01, **Figure 6A, 6B** and** 6C**). For example, the tumor volume of the PRDX6 overexpression group reached approximately 1600 mm^3^, which was significantly larger than that in the control group (only nearly 400 mm^3^). In addition, the protein levels of PCNA and PRDX6 were significantly increased in PRDX6 overexpressing SiHa cells (**Figure 7A**). Meanwhile, the results of the TUNEL test showed that PRDX6 up-regulation remarkably suppressed cell apoptosis, as implied by the decrease of the green TUNEL fluorescence (**Figure 7C**). These data indicated that the upregulation of PRDX6 promoted the proliferation, and suppressed the apoptosis of cervical cancer *in vivo*. Conversely, PRDX6 knockdown significantly inhibited tumor growth (*P* < 0.01, **Figure 6D, 6E** and **6F**), decreased PCNA protein expression, and enhanced green TUNEL fluorescence, which demonstrated that downregulation of PRDX6 suppressed the proliferation, and promoted the apoptosis of cervical cancer *in vivo* (**Figure 7B** and **7D**). The results were consistent with the effects of PRDX6 on cultured cervical cancer cells *in vitro*, which demonstrated the activity of promoting cancer progression of PRDX6* in vitro* and *in vivo*.

## Discussion

Despite many important advances in the prevention of cervical cancer, including HPV vaccines and other treatment methods, promising novel biomarkers for the diagnosis, prognosis, and prediction of cervical cancer remain rather poor 21. Peroxiredoxins (PRDXs) are a peroxidase superfamily of antioxidant enzymes that catalyze peroxide reduction, consisting of six subtypes in mammalian systems, namely, PRDX1-6. PRDXs are also a highly sensitive and specific biomarker for metastatic cancers 22-24. Among them, previous reports have demonstrated that PRDX6 is overexpressed in a variety of cancers and is involved in the tumor progression of different tumors, such as those in lung 25, thyroid 26 and colorectal cancer 16**.** Chang et al. 27 found that PRDX6 overexpression promotes the invasive phenotype of metastatic breast carcinoma. PRDX6 has also been demonstrated at higher expression level in cervical squamous cell carcinoma (CSCC) compared with normal cervical tissue by Western blot 28. These findings indicate that PRDX6 may play an important role in cervical cancer cell growth. However, the functional role of PRDX6 in the progression of cervical cancer is still unclear. Therefore, the present study aimed to investigate the expression of PRDX6 in cervical cancer compared with normal cervical tissues, and explore its effect on the biological behavior of cervical cancer cells.

In the present study, we investigated the expression level of PRDX6 in cervical cancer tissues. The results indicated that PRDX6 was significantly overexpressed in cervical cancer compared to normal cervical tissues. This result was further demonstrated in cell experiments. Furthermore, the SiHa cell line was chosen for upregulating or knocking down the expression of PRDX6 by using lentivirus transduction* in vivo* and *in vitro* to explore the biological function of PRDX6 in cell proliferation, apoptosis, migration and invasion. Among these functions, unlimited cell proliferation is the basic malignant characteristic of cancer, which may result in an alteration in the progression of the cell cycle 29**.** Schmitt et al. 30 found that PRDX6 promotes melanoma cell growth by enhancing arachidonic acid-dependent lipid signaling. Our results demonstrated that the upregulation of PRDX6 by PRDX6 lentiviruses significantly promoted the proliferation of SiHa cells, and the downregulation of PRDX6 by short hairpin RNA (shRNA) could inhibit proliferation, as shown by CCK-8 and colony formation assays. These results indicated that PRDX6 could promote cervical cancer cell proliferation. Moreover, the effect of PRDX6 on the proliferation of cervical cancer was further confirmed in cervical carcinoma xenograft model, which was consistent with our *in vitro* observations. We found that the tumors formed by SiHa cells transfected with PRDX6 overexpression adenovirus grew faster than the control Ad-RFP-infected group. The increased effect of PRDX6 was further verified by growth markers, including PCNA, in tumor tissues. However, the potential mechanism, including related signal transduction pathways, involved in the functions of PRDX6 on the proliferation and differentiation of cervical cancer cells needs to be further explored.

Apoptosis is an essential process underlying embryogenesis as well as cellular homeostasis 31. The occurrence and development of cancer is associated with abnormal proliferation and apoptosis. As a member of the superfamily of peroxidases, PRDX6 plays a vital role in the oxidative stress response, as it possesses an anti-apoptotic function. The downregulation of PRDX6 enhances sensitivity to oxidative stress 32. Pak et al. 12 demonstrated that overexpression of PRDX6 significantly suppressed the apoptosis caused by cisplatin in human ovarian cancer cells**.** Walsh et al. 33 found that knockdown of PRDX6 by transfection of siRNA in hepatoma carcinoma cells resulted in a remarkable increase in hydrogen peroxide-induced cytotoxicity through apoptosis. The present study indicated that PRDX6 plays a key role in the anti-apoptotic properties in liver cancer, and that its overexpression may be a tumor-supportive adaptation in the tumor microenvironment. In our study, PRDX6 was also found to have an inhibitory role in the apoptosis of SiHa cells, demonstrating an obvious apoptosis inhibition of cancer cells following overexpression. It has been widely recognized that members of the Bcl-2 family play a vital role in cell apoptosis 34. Among them, the Bcl-2 protein participates in cell survival and the suppression of cell death, suggesting the anti-apoptotic effect of Bcl-2. In contrast, BAX was found to promote apoptosis, which is regarded as the homologous binding partner of Bcl-2 35. As a pro-apoptotic protein, BAX is located in the outer mitochondrial membrane, and it transfers into the mitochondria during the early stages of apoptosis, indicating its importance for apoptotic signal transduction 36. Furthermore, alterations in Bcl-2 and BAX expression are a key step in determining whether cell apoptosis is induced. In the present study, the results showed that there was an increased expression of Bcl-2 and a low expression of BAX, leading to a significant elevation of the Bcl-2/BAX ratio. The opposite results were found in the PRDX6 knockdown group. Therefore, our results demonstrated that PRDX6 significantly suppresses cancer cell apoptosis via alterations in Bcl-2 family protein expression and may be related to the mitochondrial pathway.

It is widely acknowledged that migration and invasion are very important in some diseases, such as cancers 37. Tumor metastasis is considered a multistep process involving the dissemination of tumor cells to distant tissues and their successful adaptation to different tissue microenvironments. In this process, tumor cell migration is essential for invasion, and the invasion of cancer cells into surrounding normal tissue and blood vessels is the first step in tumor metastasis. Evidence indicates that PRDX6 promotes the migration and invasion of cancer cells during cancer progression. He et al. 15 found that PRDX6 promotes the migration and invasion of esophageal cancer cells. Some researchers 38 demonstrated that PRDX6 significantly stimulates the invasion of lung cancer cells, inducing the expression of urokinase-type plasminogen activator. In the present study, PRDX6 expression was closely related to the migration and invasion of cervical squamous carcinoma cells. Inversely, the invasion and migration of cancer cells with PRDX6 overexpression was remarkably attenuated by PRDX6 knockdown. These data suggested that PRDX6 might serve as a tumor promoter during the development and metastasis of cervical squamous carcinoma.

## Conclusion

In conclusion, our study illustrated that PRDX6 serves as a tumor promoter gene and results in the stimulation of proliferation, migration and invasion, as well as the suppression of apoptosis, in cervical cancer cells. Furthermore, PRDX6 enacts its carcinogenic effects by regulating Nanog, PCNA, Bcl-2 and BAX expression. Taken together, our current data provide a potential target for novel therapeutic strategies for the use of PRDX6 inhibitor, such as MJ33 (1-hexadecyl-3-(trifluoroethyl)-sn-glycero2-phosphomethanol), for preventing and controlling the development of cervical cancer in the future. However, to inhibit the occurrence and development of cervical cancer, the molecular mechanism underlying the function of PRDX6 in cervical carcinogenesis or progression needs further exploration.
